# Humic lakes with inefficient and efficient transfer of matter in planktonic food webs

**DOI:** 10.1038/s41598-023-35039-1

**Published:** 2023-05-16

**Authors:** Maciej Karpowicz, Magdalena Grabowska, Jolanta Ejsmont-Karabin, Agnieszka Ochocka

**Affiliations:** 1 Department of Hydrobiology, Faculty of Biology, University of Białystok, Ciołkowskiego 1J, 15-245 Białystok, Poland; 2 Department of Freshwater Protection, Institute of Environmental Protection-National Research Institute, Słowicza 32, 02-170 Warsaw, Poland

**Keywords:** Ecosystem ecology, Freshwater ecology

## Abstract

Humic compounds and related factors are the main constraints for the development of zooplankton in humic lakes, leading to low transfer efficiency in food webs. The results of this study indicated that some zooplankton species could have an advantage under these conditions. We found that the mass development of omnivorous *Asplanchna priodonta* in temperate humic lakes could be caused by the domination of high nutritional algae such as *Gonyostomum semen* and *Botryococcus braunii*. These algae are too large for most zooplankton to ingest, but *A. priodonta* can feed on a wide range of particles and benefit from this high-nutritional food. Small cladocerans (*Ceriodaphnia*, *Bosmina*) might be favored when picoplankton and small algae-dominate humic lakes. Therefore, some zooplankton species could have an advantage and control the development of phytoplankton, leading to the effective transfer of matter and energy in the planktonic food web in humic lakes.

## Introduction

Dystrophic (humic) lakes, according to the commonly accepted definition (after Thienemann 1925^1^), are acid lakes poor in nutrients and organisms, with brown-colored water and organic matter originating mainly from peat bogs and forests surrounding the lake^2^. Humic substances dissolved in waters acidify them and can form complexes of phosphorus, ammonia, and metal ions^3,4^; therefore, the water contains small concentrations of dissolved mineral substances. A large content of humic substances gives the brown color of the water, which increases solar energy adsorption and increases the warming of the epilimnion thus increasing thermal stratification^5–7^. As a result of sharp thermal stratification, even lakes only a few meters deep can be dimictic or meromictic with a large deficiency of oxygen below the thermocline^8^.

Although humic lakes are usually considered as unproductive^9,10^, they may contain several groups of producers. Dystrophic lakes have large numbers of bacteria that can use carbon from humic substances^11,12^. Thus, the microbiological loop seems to play a more important role than the classic trophic pyramid in harmonious (clear water) lakes^13^. The important components of primary producers in humic lakes are photoautotrophic picoplankton and mixotrophic phytoplankton. Photoautotrophic picoplankton could be favored by intensive nutrient recycling within the microbial loop^14^, while mixotrophs and osmotrophs could take advantage of the hypolimnetic pool of ammonium, high bacterial production, and low light conditions^15^. Particularly, the large flagellate *Gonyostomum semen* forms dense blooms in humic lakes at boreal and temperate latitudes^16–18^. This may explain literature reports based on studies of more than 600 freshwater lakes, showing that primary production in humic lakes may be higher than that in clear lakes^19^.

On the other hand, the high biomass of phytoplankton contrasts with the low biomass of zooplankton in dystrophic lakes, which results in low efficiency of the transfer of matter and energy in planktonic food webs^20–22^. Furthermore, dystrophic lakes are unproductive from a fishery point of view, with low fish densities or an absence of fish^23–25^. Thus, a large amount of food and low fish pressure in temperate humic lakes usually do not lead to the mass development of large-bodied zooplankton, and the limiting factors for zooplankton growth are unclear. Some authors have indicated that humic stress related to the dystrophication process is responsible for limiting zooplankton development^4,26^, and others have suggested low food quality^20^. In less productive lakes, a low diversity of available food may also intensify competition in zooplankton^27^. Our previous research suggested that a combination of factors related to the dystrophication process constrained zooplankton development. On the one hand, it could be oxygen stress, as anoxic conditions can be found 3–4 m deep, and on the other hand, high ultraviolet radiation in surface waters limits optimal conditions for zooplankton to a very narrow water layer^8^.

Zooplankton communities in temperate dystrophic lakes are generally characterized by low species richness of crustaceans and rotifers and are often dominated by one or two species (*Asplanchna priodonta*, *Ceriodaphnia quadrangula*, and *Eudiaptomus gracilis*)^25,28,29^. Moreover, very similar zooplankton communities exist in different dystrophic lakes^30^. Zooplankton biomass in open water is generally low; however, mass development of one species has been observed in humic lakes^29,31^.

During this study, we observed high zooplankton biomass in some temperate dystrophic lakes. Therefore, the main aim of this paper was to determine the combination of factors that favor the mass development of zooplankton in humic lakes. The other purpose of the study was to analyze top-down (zooplankton grazing) and bottom-up (hydrochemistry) factors that affect phytoplankton communities. Finally, we estimated the transfer of matter in planktonic food webs by comparing the phytoplankton and zooplankton biomasses.

## Results

### Environmental conditions

**Figure 1:**
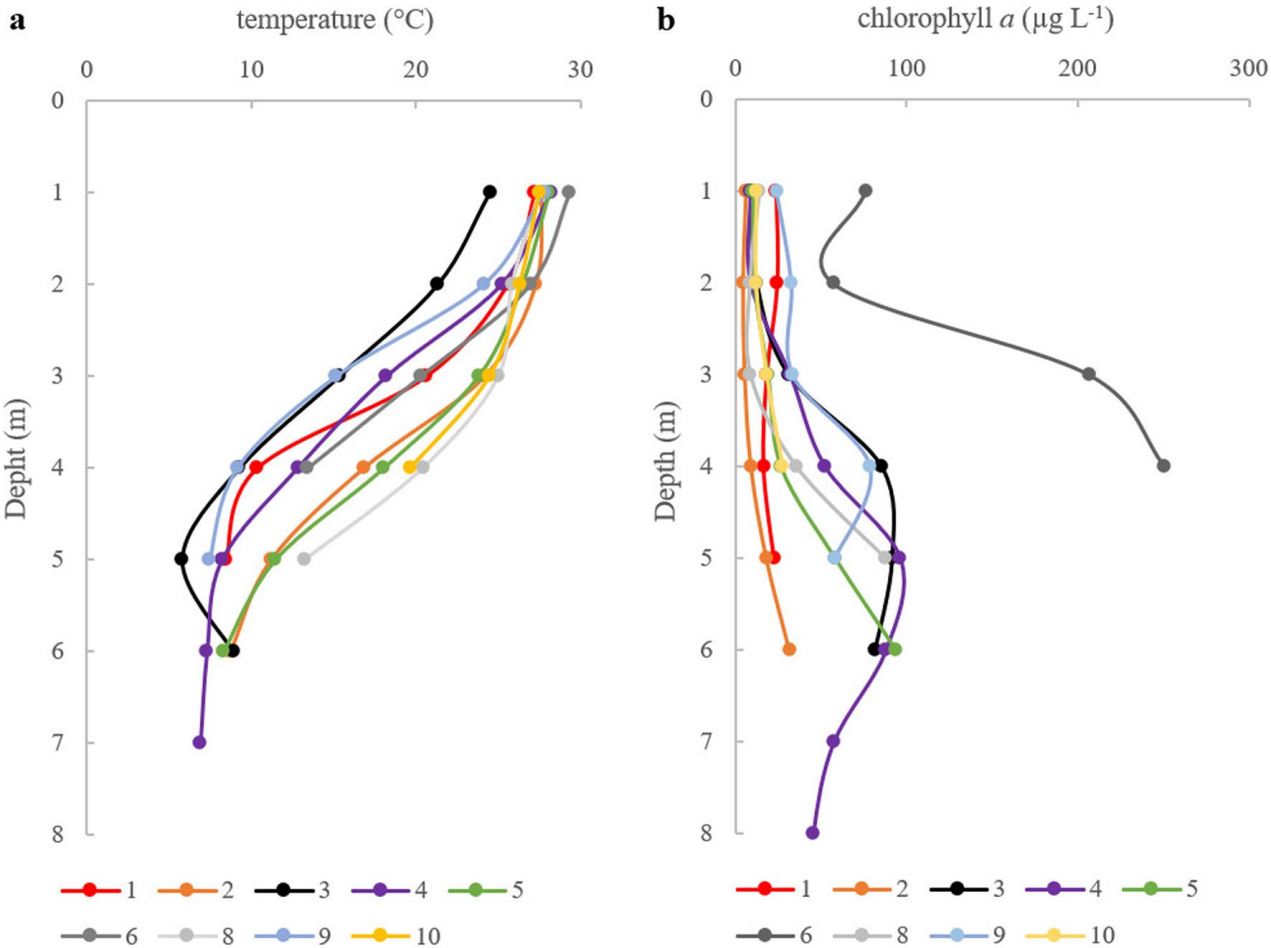
Vertical gradients of temperature (**a**) and chlorophyll *a* (**b**) in studied lakes (lake no. 7—no data).

The visibility of the Secchi disk ranged from 1.0 to 3.0 m, with the lowest values in lakes no. 6, 8, and 9 and the highest values in lakes no. 2, and 3 (Table 1). The pH ranged from 5.1 to 6.5, with an average of 5.9 ± 0.5 (Table 1). The water was characterized by a low electrical conductivity (33.3 ± 19.9 µS cm^−1^) (Table 1) and high DOC concentration (8.7 ± 2.6 mg L^−1^) (Table S1). The hydrochemical dystrophy indices (HDI) ranged from 55.5 to 74.3, with an average of 64.6 ± 5.7 (Table 1), which indicated highly dystrophic conditions. The concentration of total nitrogen was 1.30 ± 0.75 mg L^−1^, and total phosphorus was 0.46 ± 0.13 mg L^−1^ (Table S1). However, the concentrations of dissolved nutrient forms were low. The average concentration of orthophosphates was 0.64 ± 0.74 µg L^−1^, except for the epilimnion of lake no. 1, where a very high concentration of orthophosphates (162 µg L^−1^) was noticed. The average concentrations of NH_4_^+^ and NO_3_^-^ were 70 ± 15 µg L^−1^ and 30 ± 30 µg L^−1^, respectively (Table S1). We also found large differences in the concentrations of NH_4_^+^ and NO_3_^−^ in the vertical profiles and between lakes. The temperature of surface waters was high and reached up to 29.9 °C, and there were sharp temperature gradients from the surface (Fig. 1a). This resulted in very shallow epilimnion zones where the thermocline began at a depth of 1–2 m, and the temperature at 4 m was approximately 10 °C (Fig. 1a). Thermal stratification was followed by oxygen stratification. The average concentrations of oxygen in the epilimnion, metalimnion, and hypolimnion were 8.2 ± 0.6 mg L^−1^, 6.5 ± 3.2 mg L^−1^, and 1.2 ± 1.0 mg L^−1^, respectively (Table S1). There were no significant differences in nutrient forms and DOC concentrations in the vertical profiles, except for NH_4_^+^ where the highest concentrations were found in hypolimnion zones (Table S1).

**Table 1:** Characteristics of the studied dystrophic lakes in NE Poland.

Lake no	Lake name	Latitude (n)	Longitude (e)	Area (ha)	Max depth (m)	SDV (m)	pH	EC (μS cm^−1^)	HDI
1	Borkowskie	53°43′16ʺ	21°32′57ʺ	2.9	5.0	2.0	5.7	32.4	63.8
2	Tobolinka	55°01′36ʺ	23°24′23ʺ	2.6	7.5	3.0	6.5	74.2	55.5
3	Rosochaty Róg	53°45′45ʺ	21°28′42ʺ	2.2	7.5	2.7	6.1	13.3	69.6
4	Kruczek Duży	53°39′41ʺ	21°24′21ʺ	4.2	8.0	2.4	5.9	47.9	58.2
5	Suchar Dembowskich	54°02′18ʺ	23°03′33ʺ	3.1	5.5	1.8	6.4	11.7	69.1
6	Ślepe	53°53′33ʺ	23°01′46ʺ	4.8	6.5	1.0	5.3	18.1	74.3
7	Martwe	53°39′20ʺ	21°34′07ʺ	1.4	5.0	2.2	6.4	13.5	67.4
8	Sęczek	53°43′41ʺ	21°32′41ʺ	3.7	6.8	1.3	5.6	38.2	64.2
9	Konopniak	53°35′07ʺ	21°33′09ʺ	1.7	5.0	1.1	5.1	45.0	60.9
10	Suchar Wielki	54°01′40ʺ	23°03′20ʺ	11.0	8.0	2.1	5.7	38.6	62.7

*SDV* Secchi disk visibility, *EC* electrical conductivity, *HDI* Hydrochemical Dystrophy Index^4^.

### Phytoplankton communities

**Figure 2:**
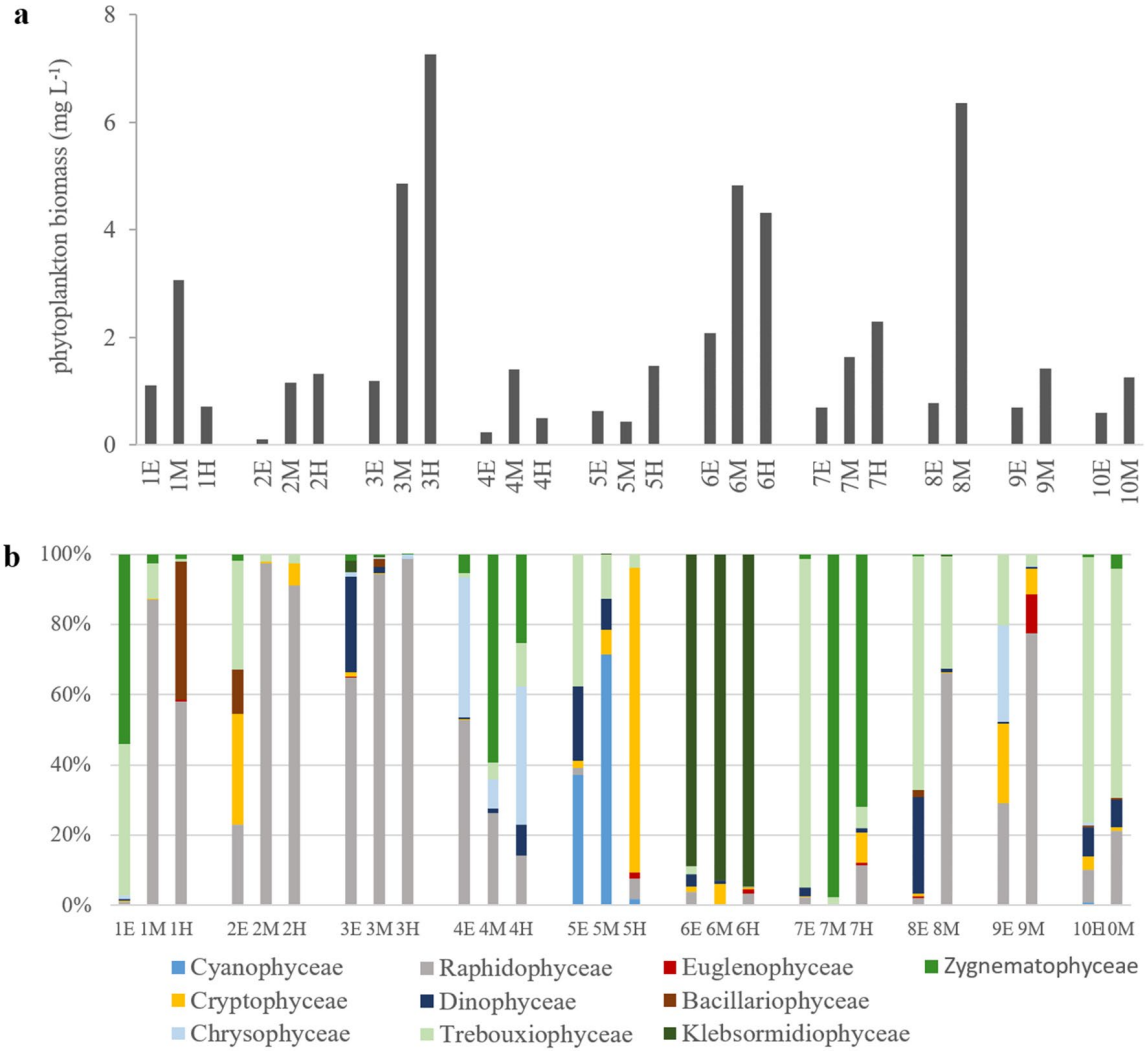
Phytoplankton biomasses (**a**), and percentage share of main phytoplankton groups (**b**) in epilimnion (E), metalimnion (M), and hypolimnion (H) of studied lakes.

**Figure 3:**
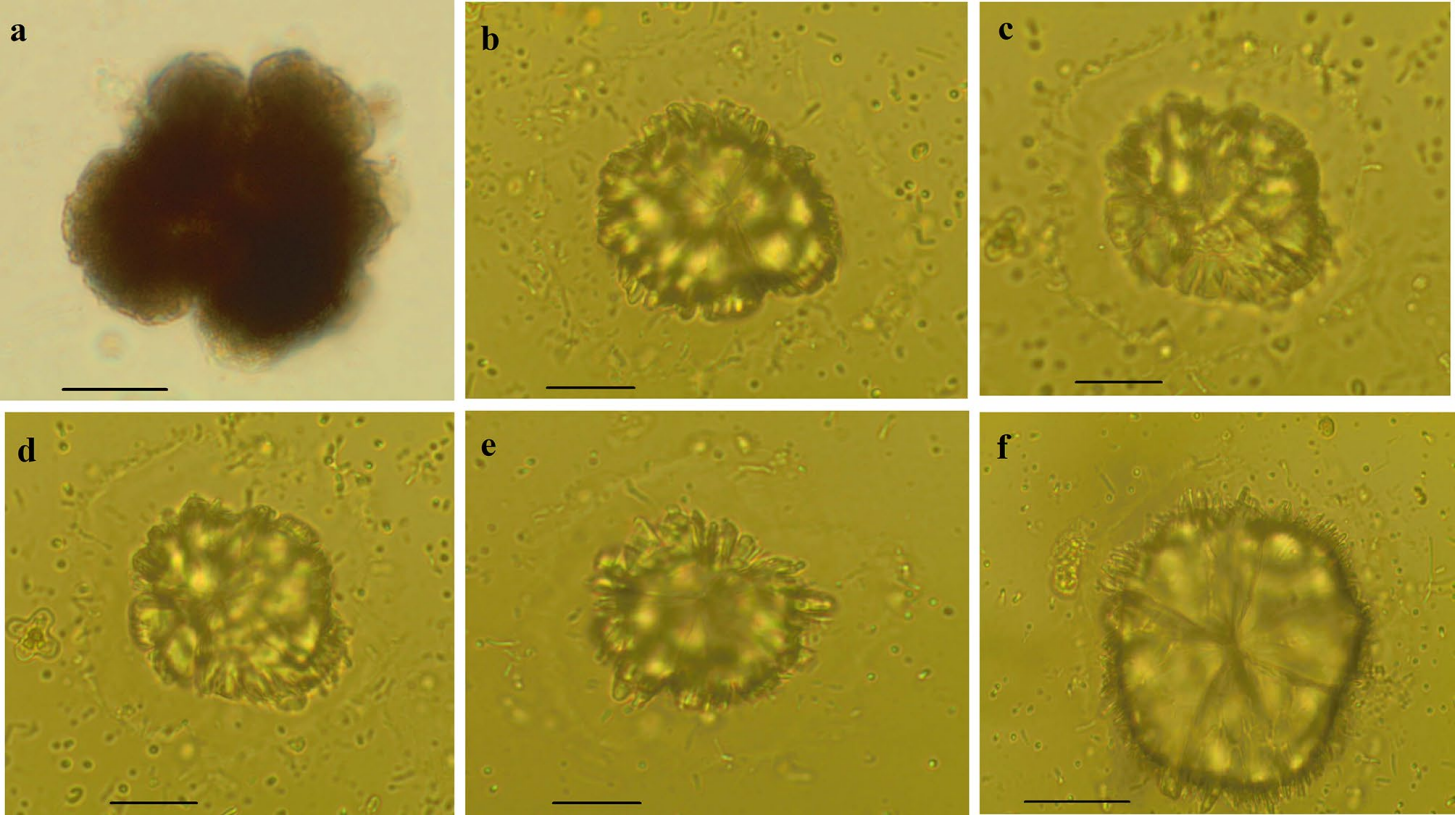
*Botryococcus braunii* from lake no. 1 (Borkowskie). (**a**) normal colony; (**b**–**f**) ‘digested’ colonies. Scale bars are 20 µm. Images of colonies fixed with Lugol's solution were taken with an inverted microscope from the Utermöhl chamber.

Phytoplankton were represented by 10 classes: Cyanophyceae, Dinophyceae, Cryptophyceae, Raphidophyceae, Chrysophyceae, Bacillariophyceae, Trebouxiophyceae, Zygnematophyceae, Euglenophyceae, and Klebsormidiophyceae (Fig. 2b). The flagellates were the most numerous group. Among them, the highest abundance reached *Cryptomonas* spp. (4661 ind. mL^−1^ in lake no. 5) (Cryptophyceae), *Mallomonas* sp. (1905 ind. mL^−1^ in lake no. 4) and *D*. *pediforme* (1029 ind. mL^−1^ in lake no. 4) (Chrysophyceae), and *Euglena* sp. (872 ind. mL^−1^ in lake no. 9) (Euglenophyceae). Cyanophyceae were determined only in four lakes no. 4, 5, 6 and 10. The most numerous Cyanophyceae was *Merismopedia tenuissima* (max. 15,805 ind. mL^−1^ in lake no. 5). Bacillariophyceae were represented only by *Asterionella formosa* in the hypolimnion of lake no. 1 (847 ind. mL^−1^). *Spondylosium papillosum* and *Cosmarium* sp. from Zygnematophyceae reached maximum abundance in the metalimnion of lake 4 (2135 ind. mL^−1^) and lake no. 7 (16,090 ind. mL^−1^), respectively. Among Trebouxiophyceae with small size the most numerous were *Stichococcus* sp. (149,760 ind. mL^−1^ lake no. 6), *Oocystis* spp. (4224 ind. mL^−1^ lake no. 9), unicellular Chlorococcales (lake no. 2, 4, 5, 9, 10—maximum 3859 ind. mL^−1^), *Crucigeniella rectangularis* (2850 ind. mL^−1^ in lake no. 10), *Gloeotila* cf. *turfosa* (2575 ind. mL^−1^ in lake no. 8), *Ankistrodesmus falcatus* (1548 ind. mL^−1^ in lake no. 7), and *Monoraphidium komarkovae* (737 ind. mL^−1^ in lake no. 5). Large colonies of *Botryococcus braunii* were found in most of the studied lakes, with the exception of lakes no. 4 and 9. In the epilimnion and metalimnion of lake no. 1, apart from a small number of typical colonies of *B. braunii* (epilimnion—24 colonies mL^−1^, metalimnion—12 colonies mL^−1^), huge numbers of ‘digested’ colonies (epilimnion—2214 colonies mL^−1^, metalimnion—1238 colonies mL^−1^) were noted (Fig. 3). Klebsormidiophyceae represented only one genus *Elakatothrix* (*E. genevensis* and *E. spirostoma* in lakes no. 1, 3, 5, 6, 8).

The total phytoplankton biomass ranged from 0.11 mg L^−1^ (epilimnion of lake no. 2) to 7.26 mg L^−1^ (hypolimnion of lake no. 3) (Fig. 2a). *Gonyostomum semen*, the only representative of the Raphidophyceae, was present in each of the lakes but strongly dominated (> 50% total phytoplankton biomass) in only 6 of them in at least one of the layers (Fig. 2b, Table S2). In lake no. 3, this species dominated all three layers, reaching from 64.9% of the total phytoplankton biomass in the epilimnion to 98.2% in the hypolimnion. In the epilimnion of lake no. 4, it constituted 52.6% of the total phytoplankton biomass; in lake no. 1 it constituted 87.1% in the metalimnion and 95.6% in the hypolimnion; and in the metalimnion of lake no. 8 and 9 it constituted 66.0% and 77.6% of the total phytoplankton biomass, respectively. Apart from Raphidophyceae, more than 50% of the total phytoplankton biomass was found among Cyanophyceae (*Merismopedia tenuissima* in the metalimnion of lake no. 5), Cryptophyceae (*Cryptomonas* spp. in the hypolimnion of lake no. 5), Trebouxiophyceae (*Stichococcus* sp. in all layers of lake no. 6, *Gloeotila* cf. *turfosa* in the epilimnion of lake no. 8, unicellular Chloroococcales in all layers of lake no. 10), Zygnemathophyceae (*Spondylosium papillosum* in the metalimnion of lake no. 4 and *Cosmarium* sp. in the epilimnion and hypolimnion of lake no. 7 (Table S2).The chlorophyll *a* concentrations ranged from 5.2 to 250 µg L^−1^, with an average of 49.8 ± 59.2 µg L^−1^. The highest chl *a* concentrations were found in lake no. 6, while in other lakes, they did not exceed 100 µg L^−1^ (Fig. 1b). The lower water layers were characterized by higher chl *a* concentrations (Fig. 1b) and higher biomasses of phytoplankton (Fig. 2a).

### Zooplankton communities

We identified 18 microcrustacean species (11 Cladocera, 5 Cyclopoida, and 2 Calanoida) (Table S3) and 29 Rotifera species (Table S4). The number of microcrustacean species in one lake ranged from 2 to 8 (Table S3), with an average of 5.2 ± 1.7 species. The number of Rotifera species in one lake ranged from 3 to 11 (Table S4), with an average of 7.4 ± 2.7 species.

**Figure 4:**
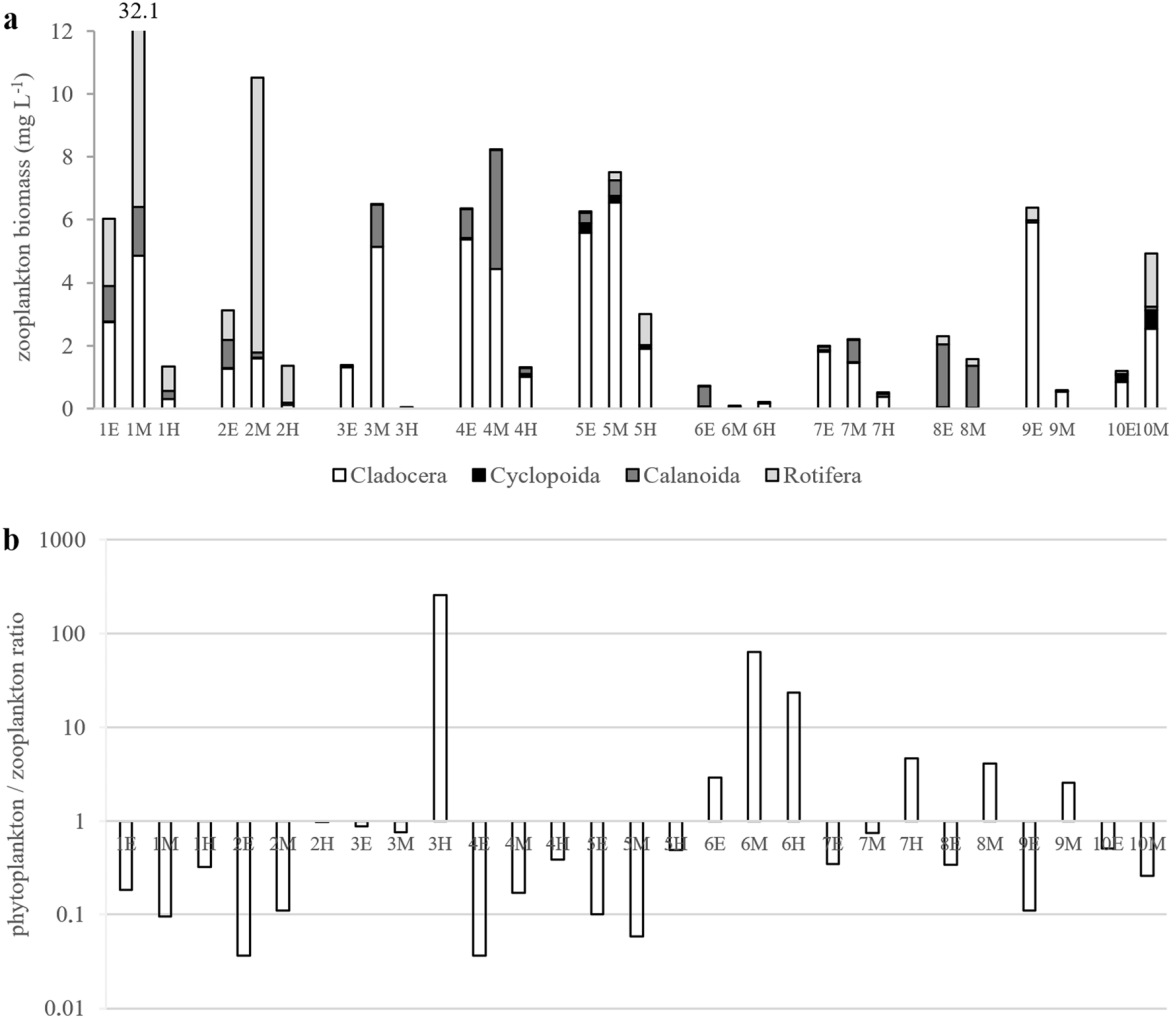
Zooplankton biomasses with a share of Cladocera, Cyclopoida, Calanoida, and Rotifera (**a**), and the ratio of phytoplankton/zooplankton (**b**) biomasses in epilimnion (E), metalimnion (M), and hypolimnion (H) of studied lakes.

Among Cladocera, *Ceriodaphnia quadrangula* was the most frequent species (all lakes except no. 6), often reaching high biomasses. *Bosmina longirostris* was found in six lakes and was the dominant component of zooplankton in lake no. 7 (Table S3). *Diaphanosoma brachyurum* was found in six lakes, reaching high biomass in lakes no. 2 and no. 3 (Table S3). Rare *Holopedium gibberum* was found in two lakes (no. 4 and no. 9), where it reached high biomasses. *Scapholeberis mucronata* and *Daphnia cucullata* were found in two lakes, while *Alonella nana*, *Eurycercus lamellatus*, *Chydorus sphaericus*, *Daphnia longispina*, and *Polyphemus pediculus* were found in single records (Table S3). The proportion of Cyclopoida in zooplankton was low in the studied lakes (Fig. 4a). The most common species were *Mesocyclops leuckarti*, found in four lakes, and *Thermocyclops crassus*, found in two lakes (Table S3). *Diacyclops bicuspidatus*, *Eucyclops* sp., and *Cyclops* sp. were single records (Table S3). Calanoida had a higher share than Cyclopoida, and they were the dominant component of zooplankton in lake no. 8 (Fig. 4a). The most common calanoid was *Eudiaptomus gracilis* (9 lakes), while *Eudiaptomus graciloides* was found only in lake no. 10 (Table S3).

The most frequent rotifer species were *Asplanchna priodonta* and *Trichocerca simoneae*, which were found in 8 lakes. *Asplanchna priodonta* achieved very high densities in the metalimnion of lake no. 1 and 2 (1204 and 916 ind. L^−1^, respectively) and dominated in three other lakes. *T. simoneae*, on the contrary, although very frequent, had much lower densities, usually lower than 10 ind. L^−1^ and up to 156 ind. L^−1^ in lake no. 9. Present in at least half of the studied lakes were *Conochiloides dossuarius*, and two species of *Polyarthra* (*P. remata* and *P. vulgaris*) (Table S4).

Zooplankton biomass ranged from 0.03 to 32.1 mg L^−1^. Higher zooplankton biomass was found in lakes no. 1, 2, 4, and 5, and the lowest biomass was found in lakes no. 6, 7, and 8 (Fig. 4a). The very high biomass of zooplankton in lakes no. 2 and no. 1 was caused by the mass development of *Asplanchna priodonta*, even to 8.7 and 25.5 mg L^−1^, respectively. Due to its large size, the presence of this species had a strong impact on the biomass of Rotifera communities in the other six lakes, while higher biomass in lakes no. 4 and no. 5 was related to the large development of *Ceriodaphnia quadrangula*, even to 4.94 and 6.39 mg L^−1^, respectively. In the vertical profiles, the highest zooplankton biomasses were found in metalimnion zones, but in some lakes, high biomasses were also observed in epilimnion zones (Fig. 4a).

### Phytoplankton–environmental relationship (bottom-up)

**Figure 5:**
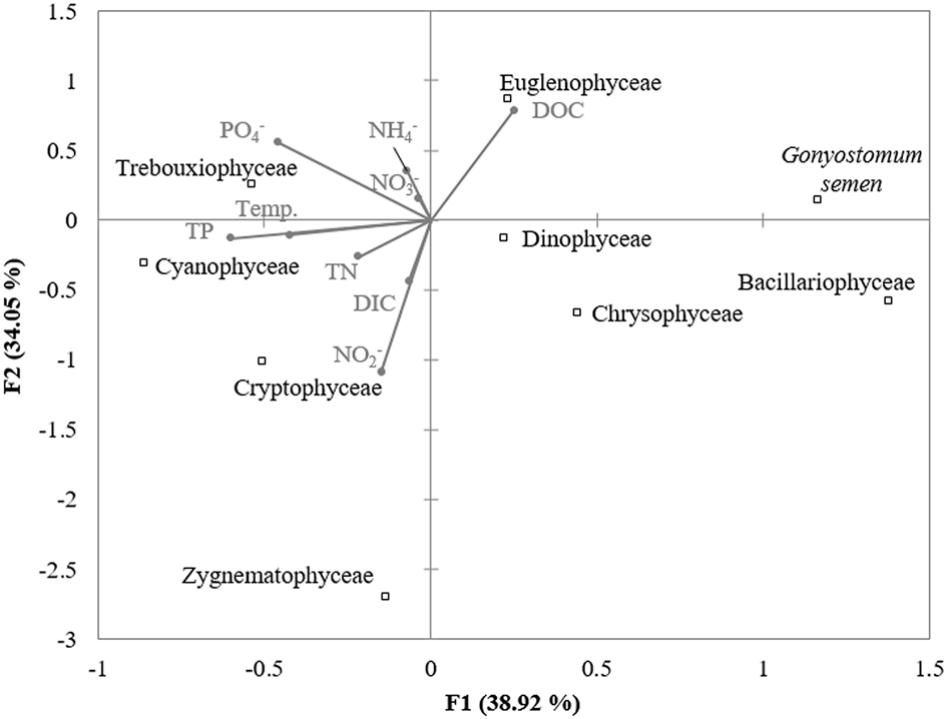
Relations between phytoplankton and physicochemical parameters of water (bottom-up) visualized by the Canonical Correspondence Analysis map.

The ANOVA results indicated a weak relationship between chl *a* concentrations and all analyzed environmental parameters (F = 1.49; p = 0.24); however, temperature could be negatively related to chl *a* (F = 4.24; p = 0.057). The dominant species, *Gonyostomum semen* could be favored by higher DOC concentrations, lower temperatures and lower nutrient concentrations (Fig. 5). Euglenophyta was strongly related to DOC (Fig. 5), while Trebouxiophyceae and Cyanophyceae were favored by higher temperatures and nutrient concentrations (Fig. 5).

### Phytoplankton–zooplankton relationship (top-down)

**Figure 6:**
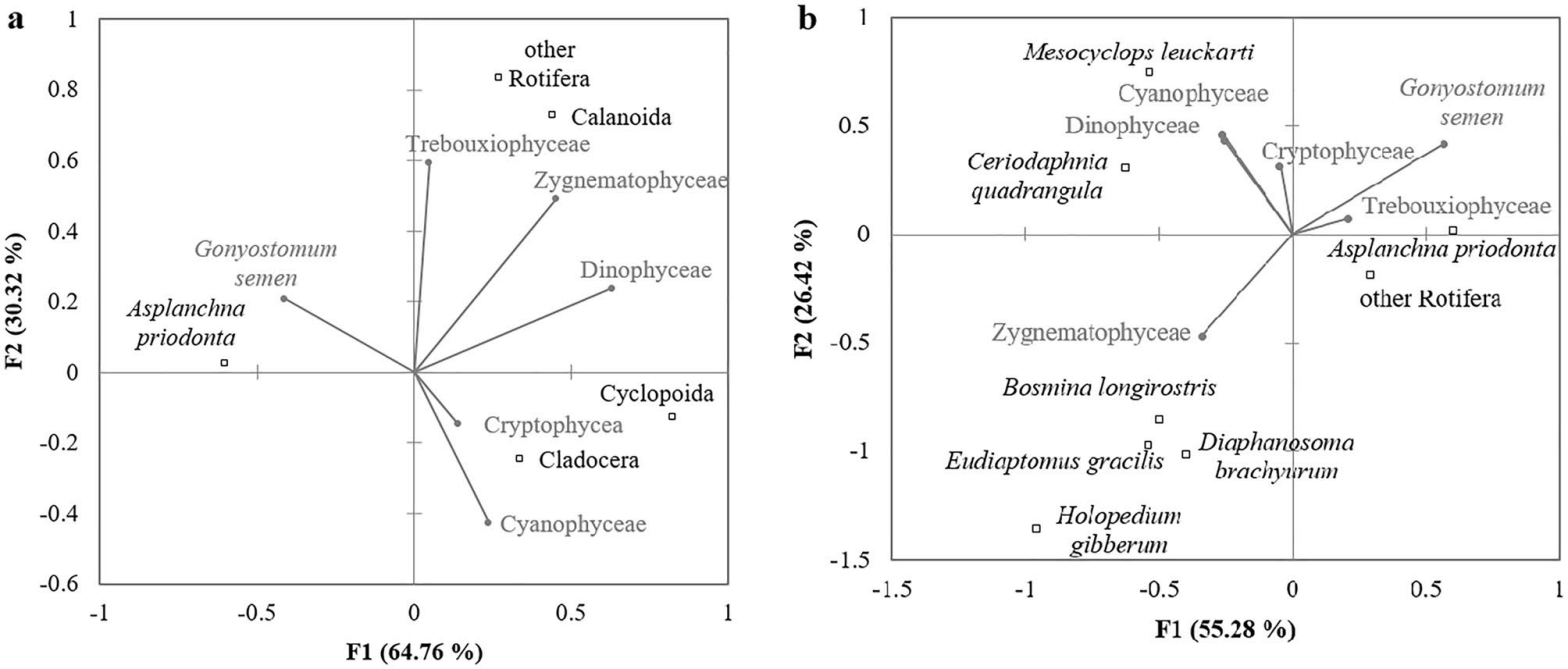
Relations between phytoplankton and zooplankton (top-down) visualized by the Canonical Correspondence Analysis map. (**a**) all lakes; (**b**) lakes with ‘effective transfer’ (higher biomass of zooplankton than phytoplankton).

The results of our study suggest that the high biomass of zooplankton in lakes 1, 2, 4, 5 and 10 could control phytoplankton growth (Fig. 4b). We suppose that the extremely high biomass of *Asplanchna priodonta* in lake no. 1 could be caused by the development of *Botryococcus braunii*. We found that the number of ‘digested’ colonies of *B. braunii* (Fig. 3) was over 5 times higher than that of normal colonies (‘digested’ colonies were not counted) in lake no. 1, which may suggest that they were ingested by *A. priodonta*. In lake no. 2, the biomass of phytoplankton also could be controlled by a large number of *A. priodonta*. Additionally, in both lakes (no. 1 and 2), one of the highest biomasses of *G. semen* seems to support the development of *A. priodonta* (Fig. 6a,b). In lakes no. 4, 5, and 10, Cladocera dominated (Fig. 4a), which could control the development of small algae (i.e., *Merismopedia tenuissima*, *Stichococcus* sp., and unicellular Chlorococcales).

The biomass of the dominant phytoplankton species, *Gonyostomum semen*, could also be related to *A. priodonta* (Fig. 6a). The biomass of other species of Rotifera and Calanoida could be related to Trebouxiophyceae and Zygnematophyceae (Fig. 6a). Cladocera and Cyclopoida seem to be related to Cryptophyceae and Cyanophyceae (Fig. 6a). We obtained similar results when analyzing lakes with efficient transfer of matter (higher biomass of zooplankton than phytoplankton) in planktonic food webs (lakes no. 1, 2, 4, 5 and 10). *Asplanchna priodonta* with other rotifers was related to *G. semen* but also Trebouxiophyceae (Fig. 6b). *Ceriodaphnia quadrangula* and *Mesocyclops leuckarti* were related to Cyanophyceae and Dinophyceae (Fig. 6b). *Bosmina longirostris*, *Diaphanosoma brachyurum*, *Holopedium gibberum*, and *Eudiaptomus gracilis* were related to Zygnematophyceae (Fig. 6b).

## Discussion

Humic lakes are traditionally viewed as unproductive lakes^9,10^ with a low transfer of energy and matter along the food chain^20,21^. Eutrophication and dystrophication similarly decrease the trophic transfer efficiency of essential substances between phytoplankton and zooplankton^20,21^. The development of large-bodied zooplankton in humic lakes is limited despite the low fish pressure and high availability of food resources (phytoplankton and bacterioplankton). The main limiting factors for zooplankton include (i) humic stress related to low pH and high dissolved organic carbon and humic substances^26^; (ii) sharp thermal and oxygen stratification and anoxic conditions from 3–4 m deep^8^; and (iii) high UV radiation in surface waters^32^. The results of this study indicated that some species may have an advantage under these specific conditions. The biomass of *Asplanchna priodonta* was positively related to the dominant algae *Gonyostomum semen*. During the last few decades, *G. semen*, has increased in abundance and distribution in brown water lakes of boreal and temperate regions and it is often the dominant component of phytoplankton in these lakes^18^. This species could be considered a valuable food source due to its high concentration of polyunsaturated fatty acids (especially EPA and C18 PUFAs), but it is too large for most zooplankton to ingest^33^. However, some species such as *Asplanchna priodonta*, *Eudiaptomus gracilis* and *Holopedium gibberum* can feed on *G. semen* at high rates^17,34^. The results of our study suggest that *A. priodonta* could have an advantage over other zooplankton species during the domination of *G. semen* in phytoplankton, while *E. gracilis* and *H. gibberum* were negatively related to *G. semen* biomass.

We found extremely high biomass of *Asplanchna* in lake no. 1, which could be the result of favorable food conditions. In addition to the high biomass of *G. semen* in this lake, we found that *Asplanchna* may selectively feed on *Botryococcus braunii*. The number of ‘digested’ colonies of *B. braunii* (Fig. 3) was over five times higher than that of normal colonies, which may suggest that they were ingested by *Asplanchna*. In this lake, we observed a very high concentration of orthophosphates in the epilimnion, which was 100 times higher than that in other lakes. *Asplanchn*a may spit out the rest of digested food items again^35^, which was confirmed by our observations. The results of Kappes et al. show a strong preference of *Asplanchna* for *Botryococcus terribilis*, which was up to 99% of the food^36^. Other results also confirm that *Asplanchna* may change dietary preferences depending on the availability of food^36–38^. *Botryococcus* seems to be a ‘superenergetic’ food due to its considerable production of lipids, notably hydrocarbons^39^. *Botryococcus braunii* contains up to 61% of hydrocarbon dry weight, while other microalgae generally contain less than 5% of hydrocarbon dry weight^40^. Most of the hydrocarbons (approximately 95%) are located outside the cells in the colony matrix of *Botryococcus*, while other algae do not accumulate lipids and hydrocarbons extracellularly but rather as part of the cell membrane systems and as oil droplets within the cytoplasm^39,41^. The excellent food conditions may explain the very high biomass of *Asplanchna* observed in lake no. 1 and the approximately tenfold prevalence of dimictic female *A. priodonta* producing resting eggs. The production of these eggs requires much energy because the females hatching from them have much more lipid droplets than those hatched from subitaneous eggs^42^.

This study indicated that in some cases, dystrophic lakes could create favorable conditions for the intense development of zooplankton; however, it is mostly of one species. Therefore, the efficiency of the transfer of matter in the planktonic food webs of humic lakes could switch from being inefficient to efficient. Previous studies indicated rather low transfer efficiency of essential substances in food webs of humic lakes^12,20,21^. The results of our study suggest that the mass development of highly nutritional algae such as *G. semen* and *B. braunii* could favor the development of omnivorous *A. priodonta*, which could feed on a wide range of particles. Furthermore, the mass development of *A. priodonta* was previously observed in dystrophic lakes^29,37^.

Zooplankton in temperate dystrophic lakes have a specific and distinctive community structure. Among the most distinguishing features are the low diversity and species richness of crustaceans and rotifers, and community structures are dominated by one or two species^29,30,43^. Humic stress and related factors cause zooplankton communities to be composed mostly of cosmopolitan and eurytopic species. Among Cladocera the most common are *Ceriodaphnia quadrangula*, *Bosmina longirostris*, and *Diaphanosoma brachyurum* in temperate humic lakes^29,44^, while in boreal lakes, *Holopedium gibberum* and *Daphnia longispina* are often dominant^31,43^. *H. gibberum* is rare in temperate regions, where it is restricted mostly to relict lakes with soft water that are poor in dissolved salts of mainly calcium and magnesium^45,46^, but we found this rare species in two studied humic lakes. Another characteristic feature of humic lakes in Central Europe is the domination of Calanoida over Cyclopoida^29,43^. The results of our study confirm the relevant share of Calanoida in zooplankton biomass, with the presence of *Eudiaptomus gracilis* and *E. graciloides*.

Ejsmont-Karabin hypothesized that poor-in-species, pelagic communities in humic lakes are unsaturated with species; thus, biotic interactions are too weak to prevent colonization of their pelagial with new species^47^. *Trichocerca simoneae* is a new species to Poland. It colonizes most humic lakes and often dominates. In our study, it had a high frequency, but low density. A probable reason for its low density may be the presence of relatively high densities of rotifers and crustaceans as *T. simoneae* density was negatively connected with densities of rotifers and crustaceans in studies by Ejsmont-Karabin^47^. On the other hand, the species is probably dependent on a high abundance of *Dinobryon* sp., which was scarce in the studied lakes (JEK unpublished visual observations).

In summary, humic stress and related factors are the main constraints on the development of zooplankton in dystrophic lakes, which leads to low transfer efficiency in food webs. However, the results of our study indicated that some zooplankton species could have an advantage in these conditions. We found that the mass development of high nutritional algae such as *Gonyostomum semen* and *Botryococcus braunii* could favor the intense development of omnivorous *Asplanchna priodonta*, which could feed on a wide range of particles and benefit from these high nutritional food resources. Under these conditions, zooplankton successfully controlled phytoplankton development, which led to the effective transfer of matter and energy in the planktonic food web.

## Methods

We analyzed 10 dystrophic (humic) lakes in NE Poland (Table 1). Five lakes were located in the Masurian Lakeland (no. 1, 3, 4, 7, 8), and five lakes were located in the Suwalki Lakeland (no. 2, 5, 6, 9, 10) among which three lakes were located in the Wigry National Park (no. 5, 9, 10). These lakes are mid-forest, usually oval, and without any outlets. All the studied lakes are surrounded by peat mosses that extend into the lakes to a considerable extent. The color of the water was brown-yellow and sometimes green. The lakes are small, and their areas range from 1.4 to 11 ha (Table 1). The maximum depth of the lakes ranged from 3.5 to 8 m (Table 1). The biomass and the number of fish species were very low in the studied lakes^25,48^. Dystrophic conditions were evaluated by the hydrochemical dystrophy index (HDI), which uses data on surface water pH, electric conductivity, and DIC/DOC ratio^4^.

The sampling was conducted in the middle of the summer stagnation (07–14.07.2021). Sampling stations were located close to the deepest point of each lake. Water samples for chemical analyses and zooplankton samples were taken from the epilimnion, metalimnion, and hypolimnion (if possible) with a 5 L Limnos sampler. For zooplankton samples, ten liters of water were filtered through a plankton net with a 50 µm mesh size and fixed with 4% formalin. Water samples for phytoplankton analysis were fixed with Lugol solution. The field measurements included the Secchi disc visibility (SDV), conductivity (EC), pH, and dissolved oxygen by the HQ40D Multi Meter (Hach-Lange GmbH, Germany). Chlorophyll *a* (chl *a*) concentrations and temperature were measured in situ by a submersible spectrofluorometer (FluoroProbe, bbe Moldaenke, Germany). Temperature measurements taken every few centimeters allowed us to determine the water layers for sampling^49^. Chemical analyses of the water samples were conducted in the laboratory immediately after collection. The concentrations of ions (PO_4_^3−^, NH_4_^+^, NO_3_^−^, NO_2_^−^) were determined using a Dionex ICS 1100 ion chromatograph. The analyses of total phosphorus (TP) were conducted in the laboratory according to the conventional photocolorimetric method^50^. The concentrations of total nitrogen (TN), dissolved organic carbon (DOC) and dissolved inorganic carbon (DIC) were analyzed via high-temperature catalytic combustion using a TOC-L Series (Shimadzu, Japan).

Rotifers and crustaceans were determined to the species level and counted in the whole samples. Additionally, 10 length measurements for each species were made. Mean values of the animal length were used to estimate the wet weight of planktonic crustaceans by applying the equations from^51^ and for rotifers from^52^. Phytoplankton abundance was determined according to the Utermöhl method^53^. Cells, colonies, and filaments were counted. The biomass of the phytoplankton species was determined based on cell sizes and their approximations to simple geometric shapes^54^.

The relationship between zooplankton biomass and phytoplankton biomass can provide insight into the structure and function of lake biological communities^55,56^. Therefore, we used the ratio of phytoplankton biomass to zooplankton biomass to estimate the transfer of matter in planktonic food webs. Lakes with a higher biomass of zooplankton than phytoplankton in the whole profile were considered lakes with effective transfer of matter in planktonic food webs^22,55^. One-way analysis of variance (ANOVA) with type III sums of squares was used to test all pairwise differences between means. Canonical correspondence analysis (CCA) was performed to analyze the summarized effect of environmental factors on plankton communities (including vertical variability: epilimnion, metalimnion, and hypolimnion). Statistical analyses were performed with XLSTAT Ecology (Addinsoft).

## Supplementary Information

Supplementary Tables.

## Data Availability

All data generated or analyzed during this study are included in this published article and its supplementary information files (species list and raw data).
