# Supplementary material for: Humic lakes with inefficient and efficient transfer of matter in planktonic food webs
Source: Sci Rep. 2023 May 16;13:7913. doi: 10.1038/s41598-023-35039-1 (PMC10188500; doi:10.1038/s41598-023-35039-1)
Supplement: Supplementary file 1 — Supplementary Tables. [file 41598_2023_35039_MOESM1_ESM.pdf]

Supplementary Informations

for the article 'Humic lakes with inefficient and efficient transfer of matter in planktonic food webs' by Maciej Karpowicz, Magdalena Grabowska, Jolanta Ejsmont-Karabin, Agnieszka Ochocka.

Table S1. Hydrochemical parameters in the water column (E – epilimnion; M – metalimnion; H – hypolimnion) of the studied humic lakes.

lakes	layer	Temp. [°C]	O_2 [mg L^{-1}]	DOC [mg L^{-1}]	DIC [mg L^{-1}]	Color [mg Pt L^{-1}]	TP [mg L^{-1}]	PO_4^{3-} [µg L^{-1}]	TN [mg L^{-1}]	NH_4^+ [µg L^{-1}]	NO_3^- [µg L^{-1}]	NO_2^- [µg L^{-1}]
1	E	24.7	8.62	7.33	7.60	107	0.42	162.31	0.73	0.39	7.34	0.53
1	M	15.1	6.41	7.43	5.30	56.1	0.50	0.06	1.01	80.22	3.40	0.21
1	H	12.2	2.99	7.04	8.45	76.4	0.44	0.05	0.76	2.47	4.81	0.24
2	E	26.3	7.44	8.49	5.08	51.0	0.54	2.47	0.88	19.95	119.89	5.24
2	M	15.6	4.21	7.78	4.81	61.1	0.47	1.88	2.00	63.93	56.66	0.31
2	H	8.6	0.36	7.63	9.02	66.2	0.44	0.96	2.02	625.24	4.49	0.51
3	E	22.1	9.32	6.88	5.23	8.6	0.33	0.02	0.63	26.42	0.84	0.04
3	M	15	7.61	8.08	11.59	56.1	0.18	0.02	0.69	24.04	0.50	0.06
3	H	12.1	0.44	7.03	5.46	40.8	0.30	0.09	0.71	7.42	0.26	0.14
4	E	24.4	8.61	5.91	11.41	30.6	0.26	0.05	0.91	16.80	4.38	0.36
4	M	22.8	5.44	6.51	6.74	51.0	0.43	0.24	0.95	17.97	2.23	0.08
4	H	11	1.15	6.07	5.15	45.9	0.38	0.16	0.79	30.47	4.60	0.11
5	E	25.1	8.81	7.00	5.69	20.4	0.62	0.74	1.09	42.47	17.03	0.43
5	M	18.3	11.19	6.58	4.49	25.5	0.59	0.90	1.36	38.21	86.06	0.40
5	H	11	1.19	6.14	7.83	30.6	0.53	0.60	2.74	454.95	15.78	1.32
6	E	27.8	8.24	14.83	4.83	30.6	0.46	0.56	3.65	3.57	35.45	1.88
6	M	17.7	3.57	10.50	4.71	66.2	0.70	1.52	2.44	6.76	25.27	0.72
6	H	12.2	0	11.17	6.00	112.1	0.60	1.22	2.05	344.11	44.97	2.38
7	E	25.1	7.4	9.90	7.38	40.8	0.30	0.01	0.93	18.62	56.23	0.59
7	M	20.8	11.7	8.58	5.17	107.0	0.36	0.77	1.10	115.73	51.58	6.53
7	H	13.4	2.07	8.69	13.39	71.3	0.49	0.09	1.54	1.32	83.83	2.96
8	E	25.1	8.32	9.28	8.54	61.1	0.39	0.08	0.87	1.44	3.53	0.05
8	M	21.1	4.87	11.19	10.95	101.9	0.35	0.05	1.12	1.69	0.84	0.04
9	E	25.3	7.83	14.74	4.22	152.9	0.62	0.23	1.08	32.08	59.50	4.99
9	M	14.9	1.16	13.85	4.22	137.6	0.61	0.20	1.12	30.63	2.88	0.94
10	E	27.5	7.81	7.37	8.30	35.7	0.59	1.61	0.78	1.06	6.07	0.35
10	M	23.5	8.99	7.75	7.24	45.9	0.59	2.05	0.92	0.84	3.67	0.15

Table S2. The list of the dominant phytoplankton taxa with their biomass [mg L^{-1}] in vertical profiles of studied humic lakes; *italics* - for species constituting 5-10 % of total biomass; normal font - for species constituting >10-50% of the total biomass; **bold** - for species constituting >50 % of total biomass. E – epilimnion; M – metalimnion; H – hypolimnion.

lake	layer	*Merismopedia tenuissima*	*Gonyostomum semen*	*Euglena* sp.	*Cryptomonas* spp.	*Gymnodinium* spp.	*Peridinium incospicum*	*Peridinium* sp.	*Asterionella formosa*	*Dinobryon pediforme*	*Mallomonas* sp.	*Ankistrodesmus falcatus*	*Botryococcus braunii*	*Crucigenia tetrapedia*	*Crucigeniella rectangularis*	*Dictyosphaerium* sp.	*Gloeotila* cf. *turfosa*	*Monoraphidium komarkove*	*Oocystis* spp.	*Stichococcus* sp.	unicellular Chlorococcales	*Cosmarium* sp.	*Spondylosium papillosum*	*Staurastrum* sp.
1	E												0.47										0.52	*0.08*
	M		**2.67**													*0.16*								
	H		**0.41**						0.28															
2	E		0.03		0.08								0.01						0.02					
	M		**1.13**																					
	H		**1.20**																					
3	E		**0.78**			0.32																		
	M		**4.59**																					
	H		**7.16**																					
4	E		**0.12**								0.08													
	M		0.37							*0.12*				*0.08*									**0.83**	
	H		0.07					*0.03*		0.20				*0.03*							*0.05*		0.13	
5	E	0.24				0.13										0.19					*0.04*			
	M	**0.32**	*0.04*		*0.03*	*0.04*												*0.02*						
	H		*0.09*		**1.28**																			
6	E																			**1.90**				
	M				*0.283*															**4.49**				
	H																			**4.09**				
7	E											0.12	*0.04*								**0.48**			
	M																					**1.61**		
	H		0.26		*0.20*																	**1.65**		
8	E						0.18										**0.50**							
	M			**4.19**													2.05							
9	E		0.20		0.16					0.19									0.14					
	M		**1.11**	*0.14*	*0.10*																			
10	E		*0.06*					*0.05*							0.10						**0.33**			
	M		0.26					*0.10*							0.14						**0.64**			

Table S3. Biomass of crustacean zooplankton species (mg L^{-1}) in vertical profiles of studied humic lakes. E – epilimnion; M – metalimnion; H – hypolimnion.

lake	layer	*Alonella nana*	*Bosmina longirostris*	*Eurycercus lamellatus*	*Chydorus sphaericus*	*Daphnia cucullata*	*Daphnia longispina*	*Diaphanosoma brachyurum*	*Holopedium gibberum*	*Ceriodaphnia quadrangula*	*Scapholeberis mucronata*	*Polyphemus pediculus*	*Mesocyclops leuckarti*	*Thermocyclops crassus*	*Diacyclops bicuspidatus*	*Eucyclops* sp.	*Cyclops* sp.	copepodite Cyclopoida	nauplii Cyclopoida	*Eudiaptomus gracilis*	*Eudiaptomus graciloides*	copepodite Calanoida	nauplii Calanoida
1	E		0.23					1.87		0.66				0.01						0.58		0.54	0.02
	M		0.01							4.84										1.18		0.38	0.00
	H		0.01					0.01		0.30										0.20		0.05	0.00
2	E							1.19		0.02	0.02	0.04			0.01	0.01				0.69		0.21	0.00
	M							0.86		0.74							0.01			0.12		0.05	
	H									0.12	0.01									0.05			
3	E		0.26					0.06		0.99								0.01	0.00	0.01		0.03	0.00
	M	0.02	0.18					0.02		4.70								0.00		0.95		0.37	0.00
	H		0.01							0.02													
4	E		0.01	0.14				0.02	0.25	4.94			0.03					0.02		0.37		0.55	0.00
	M		0.03						0.49	3.92										2.42		1.35	0.01
	H		0.06					0.24	0.14	0.57			0.05					0.06	0.00	0.05		0.12	0.01
5	E									5.58			0.12					0.19	0.01	0.15		0.17	0.01
	M					0.15				6.39			0.15					0.06	0.00	0.22		0.27	0.01
	H									1.91			0.05					0.05	0.00				
6	E		0.04		0.00								0.02					0.01	0.00	0.34		0.30	0.00
	M				0.00						0.01							0.00		0.05		0.01	0.00
	H		0.12		0.04															0.02		0.00	
7	E		1.52					0.02		0.26				0.01				0.03	0.01	0.10		0.02	0.00
	M		0.77							0.69				0.01					0.00	0.67		0.03	0.01
	H		0.00							0.36										0.07		0.04	0.00
8	E									0.04										0.98		0.55	0.47
	M									0.02										0.58		0.26	0.50
9	E							0.05	1.96	3.90										0.03		0.03	0.02
	M								0.17	0.38													0.00
10	E		0.14				0.06			0.64			0.12					0.09	0.00		0.02	0.01	0.01
	M		0.04			0.12	0.06			2.31			0.45					0.13	0.01		0.11	0.01	0.00

Table S4. Biomass of Rotifera species (mg L^{-1}) in vertical profiles of studied humic lakes. E – epilimnion; M – metalimnion; H – hypolimnion.

lake	layer	*Ascomorpha ecaudis*	*Ascomorpha ovalis*	*Asplanchna priodonta*	*Cephalodella tenuiseta*	*Collotheca mutabilis*	*Collotheca pelagica*	*Colurella obtusa*	*Conochiloides coenobasis*	*Conochiloides dossuarius*	*Filinia longiseta*	*Gastropus stylifer*	*Keratella cochlearis*	*Keratella quadrata*	*Lecane acus*	*Lecane bulla*	*Lecane closterocerca*	*Lecane elasma*	*Lecane flexilis*	*Lecane galeata*	*Lecane stichaea*	*Lecane tenuiseta*	*Polyarthra minor*	*Polyarthra remata*	*Polyarthra vulgaris*	*Synchaeta longipes*	*Synchaeta pectinata*	*Trichocerca cylindrica*	*Trichocerca similis*	*Trichocerca simoneae*
1	E		0.13	2.56			0.24		0.33																0.22		0.16		0.14	0.11
	M	0.10		25.52																					0.65		0.14		0.18	
	H	0.10		0.76								0.76	0.13														0.78			
2	E			0.95																				0.44						
	M			8.72																				0.24						
	H			1.16																		0.90		0.22						
3	E							0.26															0.13						0.90	
	M																							0.19						0.19
	H																							0.21						
4	E			0.17		0.15	0.21			0.11		0.39	0.25		0.16										0.12					0.11
	M									0.76		0.20	0.39												0.24					0.11
	H									0.11			0.52			0.39		0.80												0.44
5	E			0.98							0.30	0.42	0.39											0.82						0.44
	M			0.25						0.66		0.84					0.10							0.37					0.10	
	H			0.98						0.20	0.40	0.84	0.78				0.10							0.19						
6	E			0.15											0.96				0.20	0.90									0.10	0.33
	M														0.48					0.90					0.34					0.15
	H														0.32															
7	E					0.30				0.98			0.13															0.67	0.75	
	M					0.22	0.36						0.27															0.55		0.18
	H												0.16															0.14		
8	E			0.20						0.23																				
	M			0.20						0.20				0.65																0.11
9	E	0.10		0.20						0.16											0.21			0.40	0.54	0.11	0.86			0.17
	M			0.98																				0.21	0.61					0.11
10	E			0.75								0.39	0.43											0.78						0.33
	M			1.65						0.13		0.20	0.33											0.19	0.68					0.88
